# An Explanatory Model of Potential Changes in Burnout Diagnosis According to Personality Factors in Oncology Nurses

**DOI:** 10.3390/ijerph16030312

**Published:** 2019-01-24

**Authors:** Emilia I. De la Fuente-Solana, Gustavo R. Cañadas, Lucia Ramirez-Baena, Jose L. Gómez-Urquiza, Tania Ariza, Guillermo A. Cañadas-De la Fuente

**Affiliations:** 1 Brain, Mind, and Behaviour Research Center (CIMCYC), University of Granada, Campus Universitario de Cartuja S.N., 18011 Granada, Spain; edfuente@ugr.es; 2 Faculty of Educational Sciences, University of Granada, Campus Universitario de Cartuja S.N., 18011 Granada, Spain; grcanadas@ugr.es; 3 Faculty of Health Sciences, University of Granada, Avenida de la Ilustración N. 60, 18016 Granada, Spain; jlgurquiza@ugr.es (J.L.G.-U.); gacf@ugr.es (G.A.C.-D.l.F.); 4 Departament of Educational Psychology and Psychobiology, Faculty of Education, International University of La Rioja (UNIR), Avenida de la Paz N. 137, 26006 Logrono, Spain; tania.ariza.castilla@unir.net

**Keywords:** burnout, nursing, oncology, personality, risk factors

## Abstract

Burnout in a hospital oncology service takes place when there is a high level of interaction between nurses and patients. The aim of the present study is to identify models that will enable us to accurately classify a person at a given level within each of the three dimensions of burnout, according to the values presented for personality related explanatory variables, for a sample of 96 oncology nurses working in the regional health service of Andalusia (Spain). A quantitative, crosscutting, multicentre, descriptive study was designed, and for this purpose data on sociodemographic and personality variables and on the three dimensions of burnout were compiled. Three categorical-response logit ordinal models were used and the prognostic ratios for each level were obtained, with respect to every other level, according to possible changes in the explanatory variables considered. Certain personality factors are associated with one or more dimensions of burnout syndrome. Thus, nurses are more likely to develop high levels of burnout if they present high levels of neuroticism and low levels of friendliness and responsibility. Further research in this field is needed to confirm and extend these findings.

## 1. Introduction

Research has been conducted into burnout syndrome for over 40 years, and the exponential growth observed in the number of studies conducted in this field reflects its importance. As observed by Maslach and Jackson [1], this problem can have severe effects, both on individuals and on the organisations in which they work. Nevertheless, further research is needed, in order to shed light on aspects of the syndrome where controversy remains, and thus facilitate the planning of prevention or intervention programmes to alleviate the harmful effects observed [2].

Burnout was defined by Maslach and Jackson as a three-dimensional syndrome characterised by “emotional exhaustion” (EE) (referring to the sensations of physical overexertion and emotional distress that result from workers’ continuous interactions with the beneficiaries of their services), “depersonalisation” (DP) (the development of cynical attitudes and responses towards these beneficiaries), and “low level of personal accomplishment” (PA) (the loss of confidence in personal accomplishment and the presence of a negative self-concept, produced by disagreeable situations) [3]. In 2001, Maslach [4] defined burnout as a “psychological syndrome that appears in response to chronic job stress”, portraying a maladaptive syndrome related to the environment and personal coping. This form of conceptualising the syndrome is based on the Maslach Burnout Inventory (MBI), which is the most commonly-used means of assessing burnout [3].

Research into burnout, since its inception [5], has shown that this syndrome has a particular impact in occupations where there is a high level of interaction between workers and those who avail of their services. Although the problem was initially addressed in the field of social services, a growing number of professions have been identified in which symptoms arise that fit within the theoretical framework of burnout syndrome. The health professions—in particular nursing—have been the object of many studies because these occupations are at a high risk of presenting burnout [6,7].

Many studies have investigated the relationship between burnout in nurses and sociodemographic variables such as age, sex, marital status, or having children [8,9,10,11] or occupational variables such as work shift, job satisfaction, or seniority in the workplace [12,13,14]. Among the occupational variables, one that is attracting increasing attention is that of the service or unit in which the nurse works, since working in a given area involves treating particular types of patients and pathologies [9,15].

In oncology service, due to the type of pathology treated and its impact on the person presenting this disease, it is interesting to consider how working in this area might influence the development of burnout among nurses [13,16,17,18]. With respect to this service, little research has been conducted into the role played by psychological variables, such as certain personality traits, in the development of the syndrome [19]. Some authors have reported a greater propensity for burnout to develop in nurses with a type D personality [20], which is characterised, among other aspects, by highly negative affectivity and marked social inhibition. On the contrary, there is a lower propensity to develop burnout when the person in question has a resistant personality, is prepared to make commitments, favours change, and feels in control of their personal environment [21].

Studies have also analysed the influence of the five personality factors on the development of burnout syndrome [22]. Thus, some authors report that burnout is positively related to neuroticism and negatively so to extraversion [23]. Other authors have also identified this positive correlation between burnout and neuroticism and the negative relationship between the EE and DP dimensions with extraversion, openness, responsibility, and friendliness [6,22,24].

Studies have highlighted the importance of these five personality factors in the development of burnout syndrome in nurses [25]. This association is usually considered by analysing correlations between these variables, but it would also be useful to identify changes in these personality variables that might lead to nurses experiencing more severe levels of burnout. Furthermore, knowing which personality variables are of most importance in predicting these changes in the severity of the disorder, as has been achieved for other psychological variables, would be very useful in the management of hospital nursing units [19,26].

In view of these considerations, we believe it necessary to study the association between these variables and the syndrome, and to determine the impact of this relationship and the extent to which it affects those presenting burnout, according to its severity [14]. Such a study is especially important for certain services, such as oncology, where the syndrome is both present and has a severe effect [13,27,28,29].

This study has two main aims: (1) to identify explanatory models of the different levels of burnout severity, according to the five personality factors, and thus to classify the severity of the diagnosis for an individual, for each of the three dimensions of the syndrome; and (2) to quantify the weight of each personality related risk factor, in terms of the prognostic ratios for the worsening or improvement of the diagnosis, for each of the dimensions of burnout syndrome, among oncology nurses.

## 2. Materials and Methods

### 2.1. Sample and Setting

The sample consisted of 96 oncology nurses working in the Andalusian Health Service, with an average age of 45.5 years (standard deviation (SD) = 8.02) and of whom 68.8% were women.

### 2.2. Variables and Instruments

An ad hoc questionnaire was used to obtain data on two sociodemographic variables (age and sex), the three dimensions of burnout (EE, DP, and PA), and five personality variables, that were assessed using the Spanish-language version [30] of the NEO Five-Factor Inventory (NEO-FFI) [31]. These variables were neuroticism (Nm), that is, the level of emotional instability; extraversion (Ex), the level of energy and sociability; friendliness (Fr), the level of interpersonal tendencies of approach or rejection of others; responsibility (Ry), the level of self-control and self-determination; and openness (Op), the level of intellectual curiosity and aesthetic sensibility. The NEO-FFI consists of 60 items (e.g., “I’m not a person who worries much.”). Each personality factor was represented by 12 items, scored on a five-point Likert scale. The overall score for each factor was calculated as the sum of its 12 items.

The three dimensions of burnout were measured with the Spanish-language version of the Maslach Burnout Inventory (MBI) [32]. The MBI consists of 22 items: 9 for EE, 5 for DP, and 8 for PA. In order to determine low, medium, or high levels of burnout, for each dimension, we used the values proposed in the MBI handbook [3].

### 2.3. Design and Procedure

A quantitative, crosscutting, multicentre, descriptive study was performed with a sample of oncology nurses in Andalusia (Spain). The nurses were contacted with the collaboration of provincial delegations of the Spanish nursing union (SATSE), which coordinated reception of the completed questionnaires. 

### 2.4. Ethics

Participation in the study was voluntary, individual, and anonymous, and the estimated time needed to complete the questionnaire was 45 min. All participants received information on the study and gave their written informed consent. This study was approved by the Ethics Committee of the University of Granada (393/CEIH2017) and performed in accordance with the ethical standards of the Declaration of Helsinki (2013).

### 2.5. Statistical Methods

Three categorical-response logit ordinal models were used [33,34,35], one for each dimension of burnout, in which the explanatory variables were psychological and related to personality. For each model and for each dimension of the syndrome, we determined which variables favoured transitions among levels of burnout. A principal-effects model containing the effects of factors without interaction was found to best fit the data obtained. The goodness of fit was confirmed by the likelihood-ratio test and Pearson’s chi-square test. The statistical significance of the parameters in each model was assessed by the Wald test, and the prognostic ratios of each level were obtained in relation to the other levels, according to possible changes in the explanatory variables considered. All analyses were performed using the SPSS 19.0 statistical program (SPSS Inc., Chicago, IL, USA).

## 3. Results

### 3.1. Description of the Sample and Levels of the Three Dimensions of Burnout

Table 1 shows the mean values and standard deviations of the personality factors considered.

The scores obtained for the three dimensions of burnout syndrome were categorised as low, medium, or high, in accordance with the indications of the MBI handbook. It was observed that 18.8% of the participants presented high EE, while 39.6% had a medium score for this dimension. Additionally, 20.8% of the nurses scored highly for DP and 34.4% of them had a medium score in this respect. For PA, 45.8% obtained a low score and 25% had a medium score. The prevalence of these low, medium, and high scores and the descriptive values for each of the dimensions of burnout are shown in Table 2.

### 3.2. Explanatory Models for Each of the Dimensions of Burnout

The model obtained for each dimension of burnout includes the five explanatory variables related to personality, and takes the following form:

(1) $${\hat{L}}_{s}\left(Nm,Fr,Ry,Ex,Op\right)={\hat{B}}_{0s}-{\hat{B}}_{\mathrm{Nm}}Nm-{\hat{B}}_{\mathrm{Fr}}Fr-{\hat{B}}_{\mathrm{Ry}}Ry-{\hat{B}}_{Ex}Ex-{\hat{B}}_{\mathrm{Op}}Op;\;s=1,2$$

The parameters obtained for each of the explanatory variables in the ordinal logistic regression model, for each dimension of burnout, are shown in Table 3, Table 4 and Table 5.

The log-likelihood test for each model produced the following results: χ^2^(5, *N* = 96) = 22.072, *p* = 0.001 < 0.5 for emotional exhaustion (EE); χ^2^(5, *N* = 96) = 36.319, *p* = 0.000 < 0.5 for depersonalisation (DP); and χ^2^(5, *N* = 96) = 58.999, *p* = 0.000 < 0.5 for personal accomplishment (PA). When these variables were included in the three models, the fit improved significantly compared to a model that only takes the constant into account. The following results were obtained by the Pearson chi-square goodness-of-fit test for each model: χ^2^(183, *N* = 96) = 200.979, *p* = 0.1172 > 0.5 for EE; χ^2^(183, *N* = 96) = 184.923, *p* = 0.446 > 0.5 for DP; and χ^2^(183, *N* = 96) = 185.539, *p* = 0.434 > 0.5 for PA. We concluded, therefore, that the model produced a good populational fit with the three dimensions of burnout.

The results obtained by the Wald test for each predictor in each dimension (see Table 3, Table 4 and Table 5) indicated that the variable Nm is significant at the population level for the EE dimension, that Fr is significant for DP and PA, and that Ry is significant for PA. Table 3, Table 4 and Table 5 also show the odds ratios for increasing or decreasing severity in the variables found to be significant in each of the models. When there were unit increases in these variables, the odds ratios were significant. If Nm increased by 6 units, the odds ratio of moving to a high level of EE was 1.736 times greater than it would have been if Nm did not increase. A similar effect, but in the opposite direction, took place with Fr in DP. Thus, if Fr decreased by 6 units, the odds ratio of passing to a high level of DP was 3.073 times greater than it would be otherwise; on the contrary, with an increase of 6 units in Fr, the odds ratio of passing to a low PA was 3.217 times greater than when no such increase occurred.

Finally, in the PA dimension, following a decrease of 6 units in the value of Ry, the odds ratio of passing to a low level of PA was 3.217 times greater than when there was no such decrease in Ry. None of the confidence intervals of the odds ratios for changes of 6 units in the significant variables contained the value 1 (see Table 3, Table 4 and Table 5).

## 4. Discussion

The aim of this study was to generate models enabling us to classify an individual in terms of the three dimensions of burnout, according to the scores produced for personality-related explanatory variables, for a sample of nurses working in a hospital oncology service. The results obtained showed that some of these variables were significant risk factors for the prognosis of potential change to a higher or more severe level in each of the dimensions of burnout. Finally, we obtained the odds ratios with which to quantify the strength of the risk factor found for such a worsening of the prognosis.

From the results obtained, we proposed three models, one for each burnout dimension, according to the psychological variables considered. These models enabled us to predict the probability of an individual being at a burnout level of greater or lesser severity, according to changes in the explanatory variables considered, in each burnout dimension. Accordingly, we assessed the possible inclusion of these variables in a profile associated with persons with high scores for EE and DP and low ones for PA; in other words, persons with a high probability of experiencing burnout. This conclusion was drawn on the basis of having found the influence, as a risk factor, of Nm for the dimension of EE, of Fr as a protective factor in DP and PA, and of Ry as a protective factor in PA, that is, these personality factors have separate influences on the three dimensions of burnout [36].

The results showed that high values for Nm coincided with a situation of greater burnout severity in the EE dimension. Nurses who scored highly for Nm tended to perceive their surroundings in a negative light, exaggerated their problems, and viewed stimuli as threats. This outlook led them to consume resources to combat the attitude or to seek mechanisms that distracted them, instead of dealing with their workplace obligations, and, thus, significant levels of EE were provoked [36,37].

In contrast, higher values for Fr were positively associated with DP and PA. Thus, nurses who were friendly helped patients feel more comfortable in the hospital, and thus derived greater satisfaction from their work; as a consequence, their levels of DP were low [36] and their PA levels were high [37].

Finally, high values for Ry were associated with a greater likelihood of high PA. Thus, responsible nurses managed their time more efficiently, viewing this as a personal achievement, and therefore presented lower levels of DP and higher ones of PA [37].

## 5. Conclusions

In view of the above considerations, we conclude that in order to establish a risk profile for burnout among oncology nurses, personality variables should be taken into account. Nurses are more likely to develop emotional exhaustion, depersonalisation, and feelings of little personal accomplishment if they present high levels of neuroticism and low ones of friendliness and responsibility. This finding is of great interest, and can be used as a basis for designing measures to help prevent burnout among this population [8,17,38,39].

Thus, certain personality factors may influence the dimensions of burnout syndrome. In view of the considerable weight of these variables as risk or protection factors with respect to burnout, we believe further research should be conducted in this field, among populations of nurses working in oncology and other healthcare services, in order to ratify the results presented in this paper and perhaps to refine the models proposed. Furthermore, this would better establish the role played by personality factors and facilitate the creation of risk profiles for each level and dimension of burnout.

### 5.1. Study Limitations

This study presents some limitations, which should be considered when interpreting the results presented. Firstly, the design used makes it impossible to derive conclusions regarding causal relations. In future studies, a longitudinal design could be implemented to reflect the progression of the burnout process in oncology nurses. Another limitation is the use of a sample that was nonrandomised and not very large. Nevertheless, the fact that these nurses worked in different healthcare centres counteracted this problem, to some extent, and provided a realistic panorama of health services. Finally, since some of the groups analysed were relatively small, the corresponding results should be taken with caution.

### 5.2. Clinical Implications

Oncology nurses are a risk group for burnout syndrome because of the special nature of their work and the type of patients they care for [40]. Recent research [41] and the present study confirm that the development of burnout is influenced not only by the work performed and by sociodemographic factors, but also by the nurse’s personality.

This study identified changes in personality variables that might make nurses more likely to suffer burnout and determined which personality variables bore most weight, thus enabling us to predict and characterise changes in the severity of the disorder. This type of forewarning would be very useful in the management of nursing units.

The analysis performed yields valuable information on risk and protective factors, facilitating the establishment of risk profiles for this syndrome, making it more readily diagnosable and its evolution predictable. This outcome represents a major advance in the prognosis of burnout.

Nevertheless, further research is needed on this subject, for example, expanding the focus to consider nurses working in other hospital areas [42,43].

## Figures and Tables

**Table 1:** Descriptive analysis of the variables related to personality.

Variable (*N* = 96)	M	SD
Nm	25.85	6.79
Fr	45.81	5.90
Ry	47.76	6.08
Ex	43.39	6.67
Op	39.53	6.68

Note: Nm = neuroticism, Fr = friendliness, Ry = responsibility, Ex = extraversion, Op = openness, M = mean, SD = standard deviation.

**Table 2:** Levels of burnout in each of its dimensions.

Levels of Burnout	EE			DP			PA		
	Low	Medium	High	Low	Medium	High	Low	Medium	High
%	41.6	39.6	18.8	44.8	34.4	20.8	45.8	25.0	29.2
M (SD)	16.75 (9.144)			5.64 (5.201)			36.58 (8.511)		

Note: EE = emotional exhaustion, DP = depersonalisation, PA = personal accomplishment, M = mean, SD = standard deviation.

**Table 3:** Ordinal logistic regression for the emotional exhaustion model.

Predictor	B	SD	Wald	*p*	Odds	95% CI for Odds	
						Lower	Upper
EE = 1	−3.482	2.593	1.803	0.179			
EE = 2	−1.303	2.567	0.258	0.612			
Nm	0.092	0.034	7.109	0.008	0.912	0.853	0.976
Fr	−0.079	0.044	3.211	0.073	1.082	0.993	1.180
Ry	0.019	0.042	0.203	0.652	0.981	0.905	1.065
Ex	−0.036	0.036	1.004	0.316	1.036	0.966	1.111
Op	−0.029	0.034	0.717	0.397	1.029	0.963	1.101

Note: EE = emotional exhaustion, Nm = neuroticism, Fr = friendliness, Ry = responsibility, Ex = extraversion, Op = openness, B = estimated parameter, SD = standard deviation, Wald = Wald statistic, *p* = *p*-value, Odds = odds ratio, CI = confidence interval, Lower = lower limit of the CI, Upper = upper limit of the CI.

**Table 4:** Ordinal logistic regression for the depersonalisation model.

Predictor	B	SD	Wald	*p*	Odds	95% CI for Odds	
						Lower	Upper
DP = 1	−12.340	3.057	16.297	0.000			
DP = 2	−10.246	2.958	11.996	0.001			
Nm	0.007	0.035	0.037	0.847	0.993	0.928	1.063
Fr	−0.187	0.051	13.698	0.000	1.206	1.092	1.331
Ry	−0.043	0.043	0.979	0.323	1.044	0.959	1.136
Ex	0.007	0.037	0.038	0.845	0.993	0.924	1.067
Op	−0.047	0.036	1.727	0.189	1.048	0.977	1.125

Note: DP = depersonalisation, Nm = neuroticism, Fr = friendliness, Ry = responsibility, Ex = extraversion, Op = openness, B = estimated parameter, SD = standard deviation, Wald = Wald statistic, *p* = *p*-value, Odds = odds ratio, CI = confidence interval, Lower = lower limit of the CI, Upper = upper limit of the CI.

**Table 5:** Ordinal logistic regression for the personal accomplishment model.

Predictor	B	SD	Wald	*p*	Odds	95% CI for Odds	
						Lower	Upper
PA = 1	−19.233	3.734	26.525	0.000			
PA = 2	−17.451	3.619	23.254	0.000			
Nm	0.010	0.037	0.076	0.783	0.990	0.920	1.065
Fr	−0.164	0.054	9.328	0.002	1.178	1.061	1.309
Ry	−0.195	0.050	14.982	0.000	1.215	1.101	1.341
Ex	−0.018	0.040	0.203	0.652	1.018	0.942	1.100
Op	−0.039	0.038	1.041	0.308	1.040	0.964	1.122

Note: PA = personal accomplishment, Nm = neuroticism, Fr = friendliness, Ry = responsibility, Ex = extraversion, Op = openness, B = estimated parameter, SD = standard deviation, Wald = Wald statistic, *p* = *p*-value, Odds = odds ratio, CI = confidence interval, Lower = lower limit of the CI, Upper = upper limit of the CI.
